# Adolescent, Parent and Clinician Perspectives on Time Alone in Chronic Illness Visits at a Single Children's Hospital

**DOI:** 10.1111/cch.70147

**Published:** 2025-08-05

**Authors:** Eleanor Lustig, Alfonso L. Floyd, Elizabeth A. Friedrich, Morgan Snyder, Victoria A. Miller

**Affiliations:** ^1^ Perelman School of Medicine University of Pennsylvania Philadelphia Pennsylvania USA; ^2^ Division of Adolescent Medicine Children's Hospital of Philadelphia Philadelphia Pennsylvania USA; ^3^ Department of Pediatrics, Perelman School of Medicine University of Pennsylvania Philadelphia Pennsylvania USA

**Keywords:** inflammatory bowel disease, juvenile idiopathic arthritis, sickle cell disease, transition to adult care, type 1 diabetes

## Abstract

**Background:**

Time alone between adolescents with chronic illnesses and their clinicians may contribute to patient‐centred care and transition readiness within this population. Shifts in the adolescent–parent–clinician relationship underpin this contribution; as such, this qualitative study explored adolescent, parent and clinician perspectives on time alone in routine visits for paediatric chronic illness.

**Methods:**

Semi‐structured interviews with English‐speaking adolescents (ages 12–17, *n* = 65) and their parents (*n* = 63) were conducted after follow‐up visits for inflammatory bowel disease (IBD), juvenile idiopathic arthritis (JIA), sickle cell disease (SCD) or type 1 diabetes (TID) at specialty clinics affiliated with the Children's Hospital of Philadelphia. Clinicians (*n* = 16) at the same clinics were also interviewed for this cross‐sectional qualitative study. Interviews were transcribed, and thematic analysis was performed using an inductive approach.

**Results:**

Qualitative analysis yielded six themes: (1) Clinician, parent and adolescent factors influence provision of time alone; (2) some adolescents communicate more openly with their clinician during time alone; (3) some adolescents do not share new information during time alone; (4) time alone facilitates the development of the clinician‐adolescent relationship and helps prepare the adolescent for the transition to adult care; (5) clinicians continuously re‐negotiate the parent–adolescent–clinician relationship to meet adolescents' needs; and (6) time alone can raise challenges for clinicians.

**Conclusion:**

Time alone can affect information sharing and relationship building between adolescents with chronic illnesses and their clinicians. By fostering open communication and trust in the adolescent–clinician relationship, time alone may help prepare adolescents with chronic illnesses for the transition to adult care.

## Summary

- The transition from paediatric to adult care is an important period in the trajectory of many paediatric chronic illnesses.
- Time alone between adolescents and their clinicians may provide valuable opportunities for building trust and augmenting adolescents' self‐management skills.
- Time alone is provided less routinely in chronic illness care than paediatric primary care.
- This study found that adolescents, parents and clinicians view time alone as a valuable opportunity for adolescent skill‐building in advance of the transition to adult medical care.

**Abbreviations**
IBD: inflammatory bowel disease
JIA: juvenile idiopathic arthritis
SCD: sickle cell disease
T1D: type 1 diabetes

## 1 Introduction

During adolescence, young people develop habits and health literacy skills that shape their health into adulthood (Banspach et al. 2016; Bonnie and Backes 2019; Sawyer et al. 2012). For adolescents with chronic illnesses, this process informs how they navigate the transition from dependence on caregivers to independent self‐management of their health and consequent disease‐related outcomes (van Staa et al. 2011; Miller and Harris 2012; Miller and Jawad 2019; Garvey et al. 2012; Dovey‐Pearce et al. 2005; Monaghan et al. 2013). A growing body of research suggests that active involvement of adolescents with chronic illnesses in routine clinic visits promotes the development of disease self‐management skills and supports increased treatment adherence (Miller and Jawad 2019; Dovey‐Pearce et al. 2005; Monaghan et al. 2013; Cha et al. 2022; Gilbert et al. 2014). The split‐visit model—where the adolescent receives dedicated ‘time alone’ with their clinician as part of routine visits—offers a practical strategy for increasing adolescent involvement that appears acceptable to adolescents with chronic illnesses, their parents and their clinicians (Miller et al. 2023; van Staa et al. 2015; Sawyer and Aroni 2005).

Although there is no legal basis for time alone as a practice in the United States (US), professional societies including the American Academy of Pediatrics recommend the provision of time alone during adolescent preventive care visits to increase adolescent engagement in care and information‐sharing with clinicians (Hagan et al. 2017; Ford et al. 1997; Cheng et al. 1993; Ginsburg et al. 1995; Klein et al. 1999; Ford et al. 1999; Reddy et al. 2002; Ford et al. 2004; Al‐Shimari et al. 2022). No comprehensive clinical guidelines address the use of split visits in chronic illness care, leaving the question of whether to incorporate time alone in this context to institutions and individual providers. In the US, time alone is provided less frequently in specialty care visits than in primary care (Duncan et al. 2014; Edman et al. 2010; McKee et al. 2011) despite prior qualitative research indicating that trust, confidentiality and the opportunity to have one's voice heard during medical visits are priorities for adolescents with chronic illnesses (van Staa et al. 2011; Miller et al. 2023; van Staa et al. 2015; Britto et al. 2004; Weitzman et al. 2019; Klostermann et al. 2005). By addressing adolescents' priorities and creating opportunities for involvement, time alone may both contribute to patient‐centred care and facilitate the transition to an adult model of care, including independent health management (van Staa et al. 2015).

A deeper understanding of how time alone contributes to adolescent chronic illness care can guide practice recommendations and may improve health outcomes for this population. This qualitative study explored adolescent, parent and clinician perspectives on time alone in specialty care visits for chronic illness management, with particular attention to adolescent‐parent‐clinician dynamics and information‐sharing within the visit.

## 2 Methods

### 2.1 Recruitment and Enrolment

This study was a secondary analysis of data from a study designed to develop a measure of adolescent decision‐making involvement during specialty care visits for chronic illness (Miller et al. 2023). Participants with type 1 diabetes (T1D), sickle cell disease (SCD), inflammatory bowel disease (IBD) or juvenile idiopathic arthritis (JIA) were recruited from four paediatric subspecialty clinics at the Children's Hospital of Philadelphia (CHOP) from January through August 2022. These conditions have differences in terms of prognosis and treatment, but each is life‐threatening, involves burdensome treatments and can raise challenges with respect to treatment adherence and transition to independent self‐management. There is no standard practice or policy regarding provision of time alone at CHOP or in the clinics from which we recruited participants.

Adolescent eligibility included (a) aged 12 through 17 years; (b) diagnosis of IBD (Crohn's disease, ulcerative colitis, or indeterminate colitis), T1D, JIA or SCD for at least 2 months; (c) follow‐up visit with an attending physician or advanced practice nurse at the clinic during the last month; (d) English‐speaking; (e) able to understand and complete interviews (based on parental report). Adolescents with a diagnosis of autism spectrum disorder were excluded due to differences in social communication. Parents were eligible if they attended the recent visit with the adolescent and were English‐speaking. Purposive sampling was used to ensure variability in adolescent age.

During recruitment, 173 eligible dyads were identified from clinic schedules and contacted during clinic visits, by phone or by email. From these 173 families, 69 (40%) parent–adolescent dyads enrolled in the study. Of these, 67 parents and 66 adolescents completed interviews. Several interviews were excluded from the analysis (interview completed out of study window; interview could not be recorded; participant was ineligible). The final sample included 63 parent and 65 adolescent interviews.

Clinicians were recruited from April through May 2022. Inclusion criteria included being a direct clinical care provider (physician attending or fellow; advanced practice nurse) to youth at one of the aforementioned specialty care clinics. There were no exclusion criteria for clinicians. Twenty‐two clinicians were identified as potential participants and were contacted by email. Of these clinicians, five did not respond, one declined participation and 16 (73%) clinicians enrolled and completed a semi‐structured interview. All 16 interviews were included in the analysis.

### 2.2 Procedures

The study was approved by the institutional review board at CHOP (IRB 21‐018733). After obtaining consent/permission and assent, interviews were conducted by phone and audio recorded. Parents and adolescents were interviewed separately by four members of the research team (two MPH level research staff members, one BS level research staff member and one post‐doctoral psychology fellow). Three of these interviewers also served as coders. Training included reading about qualitative research methods, reading transcripts of prior qualitative interviews, conducting a mock interview, observing an experienced interviewer conduct an interview, being observed conducting an interview and receiving feedback and receiving feedback from the senior author on the transcript of the interview.

Audio recordings were transcribed by a professional transcribing service, and transcripts were uploaded into the NVivo software (*NVivo qualitative data analysis software*, Version 10, 2012). All participants were compensated with gift cards. Interviews ranged from 10 to 56 min, with a mean of 26 min for parents, 17 min for adolescents and 35 min for clinicians.

### 2.3 Measures

#### 2.3.1 Demographics Questionnaire

Parents completed a demographics questionnaire for themselves and their adolescents, which was included to capture differences in interactions with the healthcare system based on sociocultural and economic factors. Data included parent and adolescent gender, age, race/ethnicity, highest parental educational grade, family income and family structure (two‐parent, one‐parent, other). Clinicians reported their age, race/ethnicity, years of practice (because training was completed), number of outpatient patients seen per month and weeks of inpatient service per year.

#### 2.3.2 Semi‐structured Interview

Interview questions assessed parents' and adolescents' perceptions of the adolescent's recent clinic visit, or, for clinicians, visits with adolescent patients in general. The interview guide was developed to support the goal of the larger study, which was to develop items for a measure of adolescent decision‐making involvement during clinic visits. Relevant to the current analysis, adolescents were asked, ‘Do you ever meet with this provider alone (i.e., without your parents)?’ If the adolescent responded ‘yes’, they were asked, ‘How is your communication different? Your clinician's?’ Clinicians were asked, ‘Do you ever meet with adolescents alone during clinic visits, without the parent/caregiver present?’ If they responded ‘yes’, they were asked, ‘In what ways is this beneficial? Challenging?’ Clinicians who answered ‘no’ were asked why they do not meet with adolescents alone during clinic visits. Parents were not asked specific questions about time alone.

### 2.4 Coding and Analysis

The analysed sample exceeded the minimum number of interviews usually required for thematic saturation (Guest et al. 2006; Morse and Field 1995) separately for adolescents, parents and clinicians. The first step of coding involved the researchers/research assistants (RAs) coding the interview transcripts based on the topic area addressed by each interview question (18 codes for parents and adolescents; 16 codes for clinicians). A randomly selected subset of transcripts from 12 parent‐adolescent dyads (three from each illness group) and four clinicians (one from each group) was double‐coded to ensure dependability. The average kappa across pairs of coders for the topic area of ‘time alone’ was 0.92 for parent and adolescent interviews and 0.99 for clinician interviews. A text search for related phrases^1^ was then conducted to capture additional data, as parents were not specifically asked about time alone but often discussed the topic when answering other questions. Next, coded text related to ‘time alone’ was organized by disease group and participant type and categorised into preliminary themes by three RAs. This step was inductive, whereby the RAs allowed themes to emerge from the data, rather than applying a fixed analytic scheme based on a priori theory. Preliminary themes were consolidated into broader categories encompassing data from all illness groups and individuals. Final themes were refined iteratively by two team members until a consensus was reached. The analysis indicated that thematic saturation was reached for the sample as a whole and for each condition.

## 3 Results

### 3.1 Participants

Demographic data for participants are shown in Tables 1 and 2.

**TABLE 1:** Adolescent and parent demographics.

	Adolescents (*N* = 65)	Parents (*N* = 63)
Age, mean (SD), range	14.82 (1.64), 12.13–17.60	45.90 (7.05), 32–68
Gender, % (*n*)		
Female	57% (37)	79% (50)
Male	43% (28)	21% (13)
Race, % (*n*)		
Black or African American	29% (19)	30% (19)
Asian	3% (2)	3% (2)
White	60% (39)	59% (37)
Self‐reported “other” (specified in free text response as Hispanic, biracial, mixed, or Arab/Muslim)	8% (5)	8% (5)
Hispanic ethnicity, % (*n*)		
Yes	6% (4)	6% (4)
No	94% (61)	94% (59)
Medical diagnosis		
Inflammatory bowel disease	26% (17)	—
Juvenile idiopathic arthritis	23% (15)	—
Sickle cell disease	22% (14)	—
Type 1 diabetes	29% (19)	—
Highest level of education, % (*n*)		
Completed high school	—	14% (9)
Some college/tech school	—	19% (12)
College grad	—	32% (20)
Some post‐grad	—	3% (2)
Master's, PhD, MD, law degree, etc.	—	32% (20)
Total household income last year		
Less than $19 999	—	2% (1)
$20 000–$39 999	—	10% (6)
$40 000–$59 999	—	11% (7)
$60 000–$79 999	—	11% (7)
$80 000–$99 999	—	10% (6)
More than $100 000	—	52% (33)
Refused	—	5% (3)
Family structure		
Two parents in the home	—	75% (47)
One parent in the home	—	18% (11)
Other	—	6% (4)
Refused	—	2% (1)
Relationship to child		
Parent	—	97% (61)
Aunt (legal guardian)	—	2% (1)
Grandmother (legal guardian)	—	2% (1)

**TABLE 2:** Clinician demographics.

	Clinicians (*N* = 16)
Age, mean (SD), range	44 (12.07), 31–72
Gender, % (*n*)	
Female	75% (12)
Male	25% (4)
Race, % (*n*)	
Black or African American	6% (1)
Asian	6% (1)
White	81% (13)
Self‐reported other (not further specified in free text response)	6% (1)
Hispanic ethnicity, % (*n*)	
Yes	0% (0)
No	100% (16)
Role, % (*n*)	
Attending	56% (9)
Fellow	19% (3)
Advanced Practice Nurse	25% (4)
Years in practice, median, Q1–Q3	9.5, 1–20.25
Patients seen in outpatient setting per month, median, Q1–Q3	92.5, 48–130
Weeks of inpatient service per year, median, Q1–Q3	3, 0.5–7.5

### 3.2 Qualitative Findings

The analysis yielded six themes, three of which had subthemes. Unless noted, themes capture the views of participants from all illness groups and span adolescent, parent and clinician perspectives. Table 3 includes illustrative quotes for each theme.

1 Clinician, parent, and adolescent factors influence provision of time alone:

**TABLE 3:** Illustrative quotes.

Clinician, parent and adolescent factors influence provision of time alone *Emerged from clinician, parent and adolescent data*		
Clinician factors		
Adolescent data	Parent data	Clinician data
*N/A*	*N/A*	“[I meet with adolescents alone] definitely over 16 always. But if a parent wants to stay with a patient before that, I might do the general visit and then ask the parent to step out when I'm doing the exam and that if there are things that I need to address in that time during the exam I can quickly ask them before the parent steps in.” Clinician from Haematology (#11)
Parent factors		
Adolescent data	Parent data	Clinician data
“[My mom] just asked me, as well, if I'd forgot anything, if I wanted to speak with Dr. [Last Name 1] alone to talk to her about anything more private that she—that may—she might not want to be in the room for.” 17‐year‐old with SCD (#112)	“In the past, the discussion was more with the parents. And with [Child's name] transitioning, I really wanted it to be with her. I actually felt like I was going to have to say something to [Clinician's Name]…I was going to be like, maybe I will not come in the room until the end so that conversation is happening. But it was actually, this visit, it happened naturally between the two and it did not seem, like, I did not have to bring up that recommendation.” Parent of 15‐year‐old with T1D (#41)	“I have occasionally given the kids time without their parents but it's almost [always] when they have specifically ask[ed] for it, or where just the parents cannot stop talking.” Clinician from Endocrinology (#2)
	“I'll say, even, to [Child's Name], do you want me to step out? Do you want to talk to [Clinician's Name] about anything in private? That's fine, you can. I think parents need to support that, too. Respect the kid's privacy.” Parent of 16‐year‐old with IBD (#158)	*N/A*
Adolescent factors		
Adolescent data	Parent data	Clinician data
*N/A*	*N/A*	*“*If a teen – if I sense that a teen is having trouble, whether it's like active symptoms, or mood issues related to diagnosis, oftentimes we can ask the parent to leave.” Clinician from Gastroenterology (#15)
2 Some adolescents communicate more openly with their clinician during time alone and might share information they are not comfortable sharing in front of parents *Emerged from clinician, parent and adolescent data*		
2a) Some adolescents are more open to discussing sensitive topics one‐on‐one, including those relevant to their illness management		
Adolescent data	Parent data	Clinician data
“I feel like [adolescents] should have some – at least a little bit of alone time with the doctor so they can express their feelings. Because some teenagers, they are actually not comfortable with their parent being in the room with them because they do not feel like they can speak their minds.” 16‐year‐old with SCD (#17)	“Sometimes kids get a little nervous answering certain questions in front of their parents because they do not know how the parents will react if they see something that they may…not like or something like that.” Parent of 12‐year‐old with T1D (#105)	“I think the teens are much more willing to be honest and open about some of those confidential questions that we ask, like drinking, when their parents are not in the room. And that's important, just because it's risky to drink alcohol with type one diabetes. You can have dangerous lows overnight.” Clinician from Endocrinology (#1)
“I also feel like a lot of judgement left the room [when my parents stepped out], you know what I mean? Like I felt like I could be open and honest about how I felt.” 15‐year‐old with JIA (#157)	“I was glad that he asked me to leave, so she could freely open up, if there was anything that she could not say with me there.” Parent of 15‐year‐old with JIA (#53)	
“I feel like getting the parent out of the room, and then having a one on one, I feel like that would always help. Because sometimes kids feel like—they know that the doctor is there to address any issues, and sometimes they feel like they cannot do that with the parents around. So I feel like that really helps.” 15‐year‐old with JIA (#53)		
2b) During time alone, clinicians and adolescents can have an open conversation about their treatment plan, including any adherence challenges		
Adolescent data	Parent data	Clinician data
“Well, at the time, it was just Dr. [Last Name 2] in the room. So after like my dad said what he wanted to say [about medication side effects], Dr. [Last Name 2] asked him to like leave so we could like talk about it.” 15‐year‐old with JIA (#157)	“In terms of like day‐to‐day management of [her condition], talking to her directly is the way to do it.” Parent of 15‐year‐old with JIA (#157)	“I always do my best, especially in the adolescent population, to have at least a few minutes of alone time to go through more aspects of the social history, or if I get a sense that there's not full compliance going on, just trying to have a direct conversation with them about what may be behind that, and also what concerns they may have about the current plan. Things that they are just not willing to bring up in front of their parents necessarily.” Clinician from Gastroenterology (#19)
3 Some adolescents do not communicate more openly with their clinician during time alone, and do not share new information *Emerged from clinician, parent and adolescent data*		
Adolescent data	Parent data	Clinician data
*N/A*	*N/A*	“Certainly, there are other times where I have the family leave and the patient has literally nothing else to say, which is fine. I think it's nice to just give that opportunity just in case.” Clinician from Gastroenterology (#20)
3a) Some adolescents are less comfortable communicating with their clinician without their parents present		
Adolescent data	Parent data	Clinician data
“I felt more comfortable [being involved in the visit] because my mom was there and I wasn't all alone, and if I felt uncomfortable, my mom would take over.” 12‐year‐old with JIA (#52)	“[The clinician] said, oh you can go and I'll get started with him. But I know him, I know he's not gonna say so much. He′s definitely going feel more comfortable with me being there.” Parent of 14‐year‐old with T1D (#59)	*N/A*
	“I do not think that my child would respond fantastically to [time alone]. I think he prefers to have me. But even like—I do not know. Like, is there anything you want to talk about, like have Mom step out, and then, is there anything that we need to talk about that you did not want to talk about in front of your mom?” Parent of 12‐year‐old with JIA (#33)	
3b) Some adolescents do not share new information during time alone because they are comfortable sharing all relevant information in front of their parents		
Adolescent data	Parent data	Clinician data
“I find no need to separate parent and child for the visits. I think that everything is just kind of straightforward, especially for us, everything can be said to both parties.” 16‐year‐old with T1D (#67)	*N/A*	*N/A*
“I'm not one to hide things from [my family] or really keep things from them. So, just about the same amount of information that I would normally tell Dr. [Last Name 1] in the room with them, I would talk to her about outside of the room as well.” 16‐year‐old with IBD (#100)		
4 Time alone facilitates the development of the clinician‐adolescent relationship and helps prepare the adolescent for the transition to adult care *Emerged from clinician, parent and adolescent data*		
4a) Time alone strengthens the individual relationship between the adolescent and the clinician		
Adolescent data	Parent data	Clinician data
“[The doctor] would just bring up how parents can put pressure on their kids. And like how I do not have to feel that pressure when it was just us talking.” 15‐year‐old with JIA (#157)	“If I step out of the room and she just talks for four or five minutes or whatever it was, and I think that makes it easier for her [to be involved in the visit] because she knows at that point that her opinion's valuable to [the doctor].” Parent of 15‐year‐old with JIA (#157)	“I think it's helpful to establish them as an independent individual apart from their family and that my individual relationship with them is as important and more important than my relationship with their parents or their caregivers.” Clinician from Rheumatology (#8)
	“I mean I really think that portion of the visit where he talked to her alone for five minutes was a difference maker, for lack of a better term. I think that's a great way for doctors to develop a rapport with these kids.” Parent of 15‐year‐old with JIA (#157)	
4b) Parents support time alone and view it as developmentally appropriate for adolescents		
Adolescent data	Parent data	Clinician data
*N/A*	“As [my child] has matured and grown, I recognize[d] that … at a certain point during the visit, I need to step out of the room and give her a chance to talk to the doctor privately in case there was for any reason anything that she might want to talk to them about and want to talk to them not in front of me, especially once puberty hit…So I always, as she grew, started giving her the opportunity to have alone time with the doctor.” Parent of 17‐year‐old with SCD (#112)	“[Parents] appreciate, I think, me seeing…their child as independent.” Clinician from Rheumatology (#8)
	“I think the only other thing would be to not have me in the room…like have Mom step out, and then, is there anything that we need to talk about that you did not want to talk about in front of your mom? You know? I mean as teens get older, like that – that's something my pediatrician does with my teenagers, which I appreciate, and I think recognizes the age, physical and mental age.” Parent of 12‐year‐old with JIA (#33)	
4c) Time alone furthers the development and preparation of adolescents for their transition to adult care		
Adolescent data	Parent data	Clinician data
Interviewer: “You said practicing; what do you mean by practicing?” Adolescent: “Well, practicing in the sense that like soon for college… I would be doing that alone with my doctor and my mom would not be there in the room, since she typically does the talking first, I would start practicing doing that now.” 17‐year‐old with SCD (#112)	“[Time alone] really helps in my opinion – helps her to gain trust and from my perspective it's beneficial because she's a little more than two years from being 18, being an adult, and at that point she's going to be—she's going to need to be able to have those conversations on her own.” Parent of 15‐year‐old with JIA (#157)	“For the most part, I think [time alone] builds a level of trust that is really important going down the road, especially moving them towards the transition [to adult care], getting at the things that are worrying them most and quite often uncovering behavioral health issues that maybe are harder to say out loud sometimes, whether or not a parent is there.” Clinician from Hematology (#14)
5 Clinicians are continuously re‐negotiating the triadic relationship to balance adolescents' increasing independence with ongoing parental involvement in their care *Emerged from clinician and parent data*		
Adolescent data	Parent data	Clinician data
*N/A*	“[Child's name]’s young on the side of making the decisions without a full group focus and I think, as they age, I have seen that there's a more concerted effort, but it feels to me that the level is appropriate now. His involvement is appropriate and the clinician's efforts to get him involved are also appropriate.” Parent of 12‐year‐old with IBD (#139)	“There are many things that they do not want to share in front of their parents, but I also think that a parent can do a good job of reminding their kids when we bring them back together, oh, did you mention this particular issue that you had last week? And often the young adult will say, no, I forgot, but we can talk about it now.” Clinician from Hematology (#14)
		“Sometimes it can be tough if the—in the situation where the parent is not super involved in helping manage their teen's chronic illness because sometimes some of the things I'm talking to or asking them to do is just kind of over their head when it comes to trouble shooting insurance issues with coverage and things like that. I usually like to try to pull the parent in and engage them for those kinds of things.” Clinician from Endocrinology (#1)
6 Time alone can raise challenges for clinicians *Emerged from clinician data*		
Adolescent data	Parent data	Clinician data
*N/A*	*N/A*	“I think really the only challenge is when time is limited. Just sort of during the hustle and bustle of a day‐to‐day clinic. And I always do my best, but it does not happen a hundred percent of the time.” Clinician from Gastroenterology (#19)
		“I think that it's awkward to ask the parents to leave, to be totally honest. Where do they go?” Clinician from Hematology (#13)
		“If they do not want to talk then it can just be very quiet, because you do not have the parent interjecting to answer on their behalf…that can be frustrating, especially if you just have a sense that there is something wrong and they will not share it.” Clinician from Hematology (#11)

Clinicians identified that their preferences and approach to medical practice influence the provision of time alone, as do adolescent factors within the visit. Some clinicians interviewed aim to provide time alone during every visit with adolescents over a certain age, starting as early as age 14. Others only offer time alone when they infer, based on adolescent behaviours, that it would be useful. For example, if the clinician senses that the adolescent wants to share information confidentially, then they will suggest time alone. One clinician interviewed includes parents in the whole visit to facilitate openness between the adolescent and parents. Others noted using alternatives to time alone, such as asking the adolescent confidential questions during the physical exam or inviting a disease‐specific educator to speak alone with the adolescent.

Parent factors also influence the provision of time alone. Clinicians, parents and one adolescent reported that parents sometimes offer to leave the room or expedite their participation in the visit to ensure their child has time alone. Clinicians also reported implementing time alone when a parent's behaviour impedes their communication with the adolescent. For example, a clinician may ask for time alone if the parent dominates the conversation.

2 Some adolescents communicate more openly with their clinician during time alone and might share new information:

a Some adolescents are more open to discussing sensitive topics one‐on‐one, including those relevant to their illness management.

Some adolescents and clinicians expressed that time alone allows adolescents to be more forthcoming with their clinician when answering questions about sensitive topics such as mental health, substance use and sexual health. Clinicians noted that this openness allows them to gather a more accurate social history, which can be necessary to make safe medication choices. For example, information about the adolescent's sexual activity and birth control use are important if the clinician plans to start a teratogenic medication.

Parents and clinicians also noted that time alone offers adolescents an opportunity to ask the clinician questions that they are not comfortable asking in front of their parents. One parent expressed gratitude that their child was able to receive information about sensitive topics from the clinician during time alone, rather than from untrustworthy sources.

b During time alone, clinicians and adolescents can have an open conversation about their treatment plan, including any adherence challenges.

Clinicians reported that without parents present, adolescents provide more accurate self‐assessments about how they are taking their medications and may share new concerns about their treatment plan. Further, clinicians indicated that adolescents may be more forthcoming about family dynamics that influence their treatment adherence. As such, clinicians reported using time alone to discuss how adolescents are managing with their current treatment plan, especially if the adolescent is experiencing adherence challenges. Parents and adolescents also expressed that an adolescent's treatment plan and condition management is a topic discussed during time alone.

3 Some adolescents do not communicate more openly with their clinician during time alone, and do not share new information:

Parents, adolescents and clinicians noted that sometimes, adolescents do not share new information during time alone. Clinicians did not articulate why this occurs, but parents and adolescents offered insight about parent‐adolescent‐clinician dynamics which may lead to an adolescent's reticence during time alone. Parent and adolescent data comprise two subthemes.

- Some adolescents are less comfortable communicating with their clinician without their parents present.

Some adolescents reported feeling more at ease with a parent present during clinic visits. One explained that this makes them confident that they are on the same page as their parent, while another described feeling comforted that the parent could answer for them if needed. One parent mentioned that their adolescent is quiet by nature and would likely be reserved one‐on‐one with a clinician.

b Some adolescents do not share new information during time alone because they are comfortable sharing all relevant information in front of their parents.

Some adolescents shared that they are comfortable answering all questions with their parents present, so they have nothing new to say during time alone. This subtheme emerged from adolescent interviews only.

4 Time alone facilitates the development of the clinician‐adolescent relationship and helps prepare the adolescent for the transition to adult care:

- Time alone strengthens the individual relationship between the adolescent and the clinician.

Parents, clinicians and adolescents remarked that time alone helps develop the adolescent–clinician relationship. Parents and clinicians noted that it signals to the adolescent that the clinician values their opinions, building the adolescent's trust in the clinician. Adolescents did not discuss relationship‐building so directly, but several noted that clinicians communicate less formally during time alone.

b Parents support time alone and view it as developmentally appropriate for adolescents.

Parents expressed support for time alone, noting that it is age and developmentally appropriate for their adolescents to become the primary source of information in the clinical encounter. They also considered confidential conversation with the clinician to be developmentally appropriate for adolescents. Clinicians corroborated that parents generally support the practice. No adolescents commented on this topic.

c Time alone furthers the development and preparation of adolescents for their transition to adult care.

Parents and clinicians identified that time alone helps adolescents develop skills necessary to transition into adult medical care by providing opportunities for adolescents to practice self‐advocacy and navigate medical conversations alone. One clinician explained that time alone is part of a multi‐year process of increasing adolescent involvement in visits and ownership over their medical care. Adolescents also saw time alone as an opportunity for growth, identifying that it pushes them to be more involved in clinic visits.

5 Clinicians are continuously re‐negotiating the triadic relationship to balance adolescents' increasing independence with ongoing parental involvement in their care:

This theme characterises parents' and clinicians' perspectives on how the clinician–parent–adolescent relationship progresses during adolescence and the influence of time alone on this process. Adolescents did not discuss this explicitly.

Clinicians and parents noted that, while young people take on new responsibilities during adolescence, parents often continue to play an important role in their illness management. Tension can develop between the parent and clinician if a clinician's offer of time alone is seen to minimise the parent's important role. To prevent such miscommunication, clinicians take care to explain why they recommend time alone and express that they value the parent's ongoing contributions to the adolescent's care. Clinicians also observed that the degree of parental involvement in discussions about illness management and sensitive topics varies by situation. For example, if an adolescent identifies continued challenges with treatment adherence during time alone, the clinician may invite the parent back into the visit to be involved in the discussion.

6 Time alone can raise challenges for clinicians:

This theme emerged from clinician interviews only. Many clinicians shared that they would offer time alone more frequently if their schedules allotted more time per visit. Others wondered about the logistics of where parents might wait if asked to leave the room. Clinicians also cited a number of interpersonal challenges associated with time alone. Parents are sometimes caught off guard by the request for time alone, especially when adolescents are younger. Or, for adolescents who are already unhappy to be in clinic, prolonging the visit through time alone can feel like burdening the adolescent. Clinicians also expressed that it is frustrating when they provide time alone but perceive that the adolescent is still withholding information; this frustration can discourage them from providing time alone in the future. Finally, clinicians can feel conflicted when caught in the middle of parent–adolescent miscommunication and discrepant reporting, such as when an adolescent reports greater independence in their illness management than what the parent reports.

## 4 Discussion

This study suggests that adolescents, clinicians and parents find value in time alone during routine clinic visits for chronic illness care. Time alone appears to facilitate open dialogue between clinicians and adolescents around important topics and encourage adolescent independence in preparation for the transition to adult medical care, though some adolescents prefer for a parent to remain present. Clinicians identified challenges with this practice, including logistical constraints relating to clinic workflows, parent and adolescent preferences and the necessity of negotiating complex parent–adolescent dynamics.

For many adolescents, time alone leads to more open discussion about sensitive topics such as mental health, substance use and sexual health. While this aligns with research and clinical recommendations pertaining to adolescent primary care (Ford et al. 1997; Cheng et al. 1993; Ginsburg et al. 1995; Klein et al. 1999; Ford et al. 1999; Reddy et al. 2002; Ford et al. 2004), our findings illustrate the nuanced role of such information‐sharing in chronic illness management. Whereas time alone helps primary care clinicians identify, treat and counsel on issues such as substance use disorders and sexually transmitted infections (Ford et al. 1997; Ford et al. 1999; Reddy et al. 2002; Al‐Shimari et al. 2022), the present study reveals that time alone during chronic illness visits can be critical for determining appropriate treatments (e.g., considering sexual activity when prescribing teratogenic medications).

This study identified an additional sensitive topic specific to chronic illness care, which time alone may increase open communication about: treatment adherence. This topic may be particularly sensitive for adolescents who feel pressure from their parents to agree with clinicians' recommendations or to be ‘model’ patients (Beresford and Sloper 2003). Through time alone, clinicians can obtain a more accurate picture of how the adolescent manages and copes with their condition, allowing them to problem‐solve more effectively with the adolescent. The practice also allows adolescents to practice sharing this important health information before the transition to adulthood.

Some adolescents, however, do not share new information or communicate more openly one‐on‐one with the clinician. For these adolescents, parents may be facilitators of open conversation, or the presence of a parent may not impact what the adolescent shares. Adolescents who endorsed the latter stated that they do not share new information during time alone because they are already open with their parents. However, it is also possible for a parent's presence to not impact what the adolescent shares because the adolescent is withholding information from both the parent and clinician. Across Themes 2 and 3, this study found that the adolescent's openness with their parent influences whether new information is shared during time alone. Further research might interrogate how age, developmental stage and cultural or socialisation factors influence communication changes during time alone via impacts on the parent‐adolescent relationship.

Time alone in chronic illness care can serve as a bridge in an adolescent's transition to illness self‐management that allows for levels of parental support to fluctuate based on their needs. In our study, parents and clinicians shared the view that time alone is developmentally appropriate for adolescents as it offers opportunities for them to practice speaking to clinicians, advocating for themselves and navigating the medical system—key skills for a smooth transition to disease self‐management within the adult healthcare model. Clinicians reported that, after time alone, they often re‐engage parents in problem‐solving conversations regarding disease management. This finding reflects the important role parents continue to play in health management through adolescence and illustrates that split visits add value in chronic illness care by creating space for the growing independence of adolescents without curbing parental contributions to care (van Staa et al. 2015; Sawyer and Aroni 2005; Duncan et al. 2014; Klostermann et al. 2005; van Staa and On Your Own Feet Research Group 2011; Ford et al. 2011). Notably, successful integration of time alone into clinical practice requires consistent communication between the clinician, parents and adolescent about how time alone supports the adolescent's growth and transition to adult care (van Staa et al. 2015). Such communication ensures parents and adolescents alike feel that their contributions are valued throughout the transition and should be considered a precondition for implementation of routine time alone.

Overall, our results support the integration of time alone into routine chronic illness visits, given its impact on information‐sharing and skill‐building. Building on our findings, future studies could assess the impact of time alone on adolescent information sharing and adolescent‐parent‐clinician dynamics through observation of clinic visits. Quantitative measures of the impact of time alone on health outcomes after the transition to adulthood are also needed to guide clinical recommendations for split visits in chronic illness care. Recommendations regarding the frequency of time alone might depend on adolescents' age, health condition, illness severity and number of visits per year, which were not analysed in this study. We also found that clinic workflows can be a barrier for many clinicians who wish to provide time alone (McKee et al. 2011), as this practice can lengthen visits and requires somewhere for parents to wait. Thus, recommendations for regular provision of time alone should also incorporate adjustment of clinic workflows.

Several limitations affect the generalisability of this study's findings. These findings represent a secondary analysis of data drawn from a study that was not designed to assess perspectives on time alone as a primary aim. Although many parents discussed time alone during their interviews, parents were not asked about time alone in a standardized way. Adolescents, who were less forthcoming in interviews, were not probed with follow‐ups about time alone. Thus, some subthemes may be missing adolescent and/or parent perspectives that were less readily volunteered.

Study participants were drawn from a large, urban tertiary care centre in the mid‐Atlantic US. As such, findings may not be generalisable to other geographic or institutional settings. Clinician participants were majority female and non‐Hispanic white; thus, their views may not be representative of more diverse samples. Finally, to be eligible for this study, adolescent and parent participants had to be English speakers who jointly attended the adolescent's most recent clinic visit. The perspectives of English‐speaking adolescents who do not have an English‐speaking parent—and who may therefore navigate the health system more independently—were thus excluded from this analysis.

## 5 Conclusion

Adolescents, parents and clinicians found time alone valuable during routine visits for chronic illness care, including as a way to prepare the adolescent for the transition to adult care. Further research into the influence of time alone on transition preparedness, as well as the feasibility and acceptability of routine time alone in chronic illness visits, is needed to guide recommendations for clinical practice.

## Authors' Contribution

**Eleanor Lustig:** formal analysis, writing – original draft, writing – reviewing and editing. **Alfonso Floyd Jr.:** supervision, writing – reviewing and editing. **Elizabeth Friedrich:** investigation, data curation, project administration, writing – reviewing and editing. **Morgan Snyder:** investigation, data curation, writing – reviewing and editing. **Victoria Miller:** conceptualisation, methodology, investigation, resources, writing – reviewing and editing, supervision, project administration, funding acquisition.

## Ethics Statement

The study was approved by the institutional review board at Children's Hospital of Philadelphia (IRB 21‐018733).

## Consent

Written informed consent was obtained from study participants and, for patients under 18, written assent was also obtained.

## Conflict of interests

The authors have no conflicts of interest relevant to this article to disclose.

## Data Availability

Research data are not shared.

## Endnotes

^1^
‘alone’, ‘one‐on‐one’, ‘being there’, ‘directly’, ‘leave’, ‘step out’, ‘private’, ‘confidential’, ‘puberty’, ‘birth control’, ‘teratogenic’, ‘alcohol’, ‘drugs’, ‘anxiety’, ‘depression’, ‘mental health’, ‘stay’, ‘kick out’
